# Optimal activity, avalanches and criticality in a model of the Primary Visual Area

**DOI:** 10.1186/1471-2202-15-S1-P23

**Published:** 2014-07-21

**Authors:** Germano S Bortolotto, Jheniffer J Gonsalves, Mauricio Girardi-Schappo, Thiago P da Silva, Manasses P Nóbrega, Leonel T Pinto, Marcelo HR Tragtenberg

**Affiliations:** 1 Department of Physics, Federal University of Santa Catarina, 88040-900, Florianópolis, SC, Brazil; 2 Department of Chemical Engineering and Food Engineering, Federal University of Santa Catarina, 88040-900, Florianópolis, SC, Brazil

## 

The cortical processing of visual information begins at the primary visual area of the cerebral cortex (V1). It maps completely the visual field, receiving input from the Lateral Gineculate Nucleus (LGN) and transmitting the output to the secondary visual area (V2). Recently, Andreazza and Pinto have proposed a biologically motivated network of neurons in order to study in microscopic details the processing of information in V1 [1,2]. The authors discovered that the processing occurs in waves of activity, named avalanches, which travel unidirectionally inside the network layers. By analyzing the duration of the total spiking time series of these avalanches for different values of the excitatory postsynaptic potential (EPSP), the authors found a regime in which the activity lasts longer and called it the critical state of the system [3]. However, critical avalanches are generally characterized by their sizes and lifetimes being power law distributed [4-6]. The active cluster size distribution during avalanches is also another way of measuring criticality [7]. We compute these quantities and rigorously analyze the data under the critical phenomena framework to verify whether the system is critical from this point of view. The model consists of compartmental excitable neurons spatially organized as three V1 square layers of linear size *L* (namely layers 2/3, 4Cβ and 6 – the form recognition pathway). The input comes from the LGN square layer, also with linear size *L*, and the output is the last compartment of every axon coming out of layer 2/3 to connect to V2. The neurons are compartmentalized as dendrites, soma and axon. There is attenuation only when the signal is traveling along any given dendrite. The structure of the network and the parameters of the model have been chosen from experimental works [8-10]. We studied many values of EPSP and refractory period parameters, yielding regimes either with or without superposition of avalanches. We analyze each layer separately as well as the whole network statistics. The active cluster size distribution agrees with a cutoff power law fit. The cutoff of these power laws scales with the layers’ linear size, *L*. Nevertheless, contrary to our expectations, the sizes of the avalanches are distributed in a bi-modal fashion. The active cluster size power law distributions and the longest lasting activity are found for EPSP = 1.21, which is the average experimental value for V1 pyramidal neurons [11]. For other values of EPSP, both the activity vanishes quickly and the cluster size distributions lose their cutoff power law shape. Surprisingly, only the cluster size, instead of the avalanches’ sizes and lifetimes distributions, displayed power law shape. We are currently determining the behavior of an order parameter to solidify our findings.
